# Efficacy of Gefitinib and Methorexate in Patients With Advanced Stage and Recurrent Head and Neck Cancer

**DOI:** 10.7759/cureus.15451

**Published:** 2021-06-04

**Authors:** Razia Irshad, Ghulam Haider, Madiha Hashmi, Anusha Hassan

**Affiliations:** 1 Medical Oncology, Jinnah Postgraduate Medical Centre, Karachi, PAK

**Keywords:** efficacy, gefitinib, methotrexate, head and neck, cancer

## Abstract

Introduction: Gefitinib, a tyrosine kinase inhibitor (TKI), is an epidermal growth factor receptor (EGFR)-blocking drug that is effective in the treatment of lung cancer with EGFR mutations; however, its benefits for head and neck cancers are uncertain. Therefore, this study aims to determine the efficacy of gefitinib and methotrexate in patients with advanced-stage or recurrent head and neck cancer.

Methodology: Two hundred patients of age >18 years with advanced clinical stage either IVA or IVB and recurrent cases were included in this study. Patients were randomly allocated to the gefitinib (n=100) or methotrexate (n=100) group. Each patient was evaluated for demographic variables, addictions, comorbidities and history of cancer followed by clinical and radiological evaluation. Treatment response was evaluated with standard Response Evaluation Criteria in Solid Tumors (RECIST). The primary end point of the study was overall response rate (ORR). SPSS version 25 (IBM Corp., Armonk, NY, USA) was used to analyze data.

Results: Overall response of therapy was partial 11% vs 8%, stable disease 52% vs 40%, and progressive disease 33% vs 40% in gefitinib and methotrexate groups respectively. Three patients were lost to follow up in the gefitinib group and one patient in the methotrexate group. Only one death was reported in the gefitinib group and four in the methotrexate group. In recurrent cases, six patients treated with gefitinib showed partial response whereas no case of partial response was reported in the methotrexate group (27.3% vs 0%). Similarly, in the methotrexate group significantly higher numbers of progressive and stable diseases were reported for recurrent cases than in the gefitinib group (p=0.045).

Discussion: Gefitinib had marginally better results in terms of overall response and safety as compared to methotrexate, specifically in recurrent cases of head and neck cancer. This benefit for recurrent cases and ease of administration, leading to fewer hospital visits in the coronavirus disease 2019 (COVID-19) era, makes gefitinib superior to methotrexate.

## Introduction

Head and neck cancer is a leading cause of cancer deaths worldwide. The term “head and neck cancer” refers to a heterogeneous group of malignant tumors arising from the epithelial lining of the upper aerodigestive tract. The specific primary sites are subdivided by anatomic boundaries: lip and oral cavity, pharynx (nasopharynx, oropharynx, and hypopharynx), larynx, nasal cavity, and paranasal sinuses. Squamous cell cancer or its variant is the most common histologic type, accounting for 85%-95% of head and neck cancers [1]. Despite advancements in treatment, these types of carcinomas are associated with an increased risk of morbidity and mortality [2]. Global cancer statistics show a rate of 48.4% incidence and 57.3% mortality from cancer in Asian countries, with 354,864 (2.0%) new cases and 177,384 (1.9%) deaths from carcinoma of the lip and oral cavity, 177,422 (1.0%) new cases and 94,771 (1.0%) deaths from carcinoma of the larynx, 129,079 (0.7%) new cases and 72,987 (0.8%) deaths from carcinoma of the nasopharynx, 92,887 (0.5%) new cases and 51,005 (0.5%) deaths from carcinoma of the oropharynx, and 80,608 (0.4%) new cases and 34,984 (0.4%) deaths from carcinoma of the hypopharynx [3]. A recent study from 2020 of head and neck cancer epidemiology reported an increasing incidence of oropharyngeal, hypopharyngeal, lip, and oral cavity carcinomas and a decreasing incidence of larynx and nasopharyngeal carcinomas throughout the world [4]. The majority of head and neck carcinomas (∼60.0%) are reported in an advanced stage of III or IV, whereas ∼40.0% are in stage I or II. Recommendations for stage I or II management include radiotherapy or surgery, whereas stages III and IV are managed with combined modality therapy [5].

In the last few decades, the failure of therapies such as surgery, radiotherapy, or chemotherapy has been increasing, resulting in an increased incidence of recurrent carcinoma and therefore decreased survival rates. Pharmaceutical drugs with high efficacy and selectivity and low toxicity are required for the management of advanced-stage head and neck cancers [6,7], given that treatment options are limited for recurrent/advanced-stage disease, and only a few patients are suitable for surgery or re-radiation. Palliative chemotherapy is considered the standard of care for these patients, while for patients with a good Eastern Cooperative Oncology Group (ECOG) performance status (PS) [8], a platinum-based regimen with 5-FU is often considered the standard. Because most of these patients present with poor general health or prior platinum-based chemotherapy, the antimetabolite drug methotrexate is the single-agent drug of choice for a significant proportion of this patient population [9]. Various novel agents have been tested, and epidermal growth factor receptor (EGFR) inhibitors have gained particular interest. EGFR is a member of the family of tyrosine kinase receptors that is overexpressed in more than 90% of head and neck squamous cell carcinomas (HNSCC) [10]. Dysfunction of this receptor and its associated pathways tend to have significant implications for the susceptibility and prognosis of head and neck cancer [11-13].

Gefitinib, a tyrosine kinase inhibitor (TKI), is an EGFR-blocking drug that is effective in the treatment of lung cancer with EGFR mutations; however, its benefits for HNSCCs are uncertain. Furthermore, no study assessing the effectiveness of gefitinib in new and recurrent cases of HNSCCs has been performed in Pakistan. This study aimed to fill that gap by testing the effectiveness of gefitinib in the context of methotrexate in patients with advanced-stage or recurrent head and neck cancer, while underscoring the understanding that, due to differences in genetic makeup, there could be different responses within the Pakistani population. This analysis can assist in deciding which agent is the best option for these patients.

## Materials and methods

This randomized clinical trial was conducted from February 1, 2020, to July 31, 2020, at the Oncology Department of Jinnah Postgraduate Medical Centre Karachi. During the six-month study period, 200 patients with advanced-stage or recurrent head and neck cancer were enrolled in the study. Sample size was estimated using Open epi online sample size calculator by taking statistics of overall survival rate as 10% in the methotrexate group and 23.1% in the gefitinib group, power of test as 80% and 95% confidence level, the estimated sample size is 127 to 130 patients in each group. During the six-month study period, 206 patients were enrolled in the study and 54 patients were excluded. The ethical review committee of Jinnah Postgraduate Medical Centre Karachi issued approval NO.F.2-81/2019-GENL/39431/JPMC before beginning the study, and informed consent was obtained from all eligible patients before collecting data. Sociodemographic data, along with clinicopathological features and medical history, were noted on pro forma data collection paperwork [1].

The following were the criteria for the inclusion of patients in the study: (1) either gender aged >18 years; (2) biopsy confirmation of a new or recurrent case of head and neck cancer in the advanced clinical stage of either IVA or IVB; (3) normal renal, liver, and cardiopulmonary function; (4) willingness to participate in study. The following were criteria for exclusion from the study: (1) head and neck cancer in a clinical stage of I, II, or III; (2) refusal to consent; (3) medical ineligibility for chemotherapy.

A total of 206 eligible patients were randomly allocated to one group treated with methotrexate (n=103) and the other group treated with gefitinib (n=103). The computer-generated random number was to allocate patients in two arms of the study. Treatment continued until the patient was withdrawn due to unacceptable toxicity or upon the patient’s cure or death. Cancer staging was performed according to the seventh edition of the American Joint Committee of Cancer (AJCC) TNM classification. A complete history, physical examination, and clinical evaluation were conducted for each patient. Local examination and fiber optic laryngoscopy were conducted for the local extension of the tumor, as required. A computed tomography (CT) scan of the head, neck, chest, and upper abdomen was completed at baseline for primary or metastatic disease. In one group, methotrexate was intravenously administered at a dose of 40 mg/m2 per week. In the other group, gefitinib was administered orally at a dose of 250 mg or 500 mg per day. Patients in this group who were unable to swallow were allowed to dissolve the tablets in water. The primary end point of the study was overall response rate (ORR). The term overall response rate is used to describe the proportion of patients in the study who have a partial or complete response to the treatment within a certain period of time. The secondary end point of the study was disease control rate (DCR), which is often used to describe the proportion of patients exhibiting a response or stabilized disease.

Both groups were followed for six months, and the outcomes were compared. Therapy was continued until disease progression, unacceptable toxicity, or patient withdrawal. All patients were clinically and radiologically assessed before starting treatment and were also subsequently assessed clinically and, where possible, radiologically. Standard Response Evaluation Criteria in Solid Tumors (RECIST) was completed on a monthly basis to assess the patient as having a complete response, a partial response, stable disease, or progressive disease [2].

**Figure 1:**
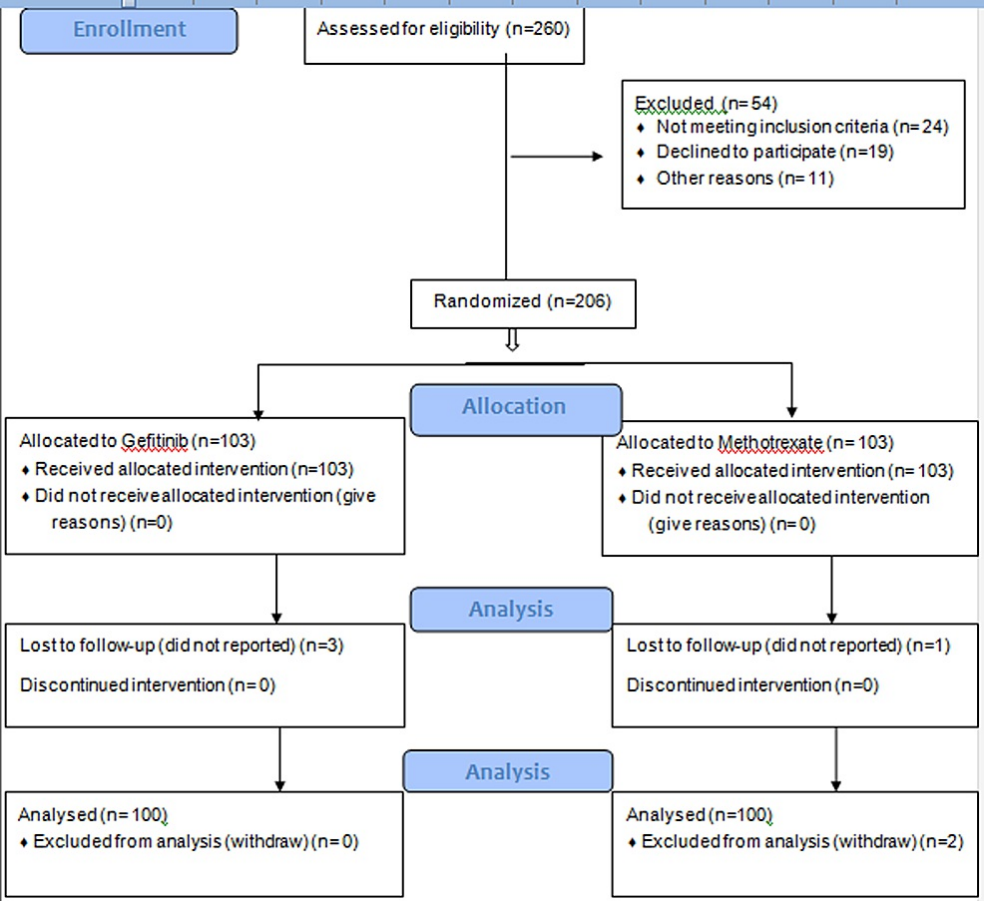
CONSORT Flow Diagram of included patients.

Data regarding socio-demographic, along with clinicopathological features and medical history, were also noted on the pre-designed questionnaire. All data were kept confidential and used for research purposes only by the principal investigator. In the final analysis a total of 200 patients were included (Figure 1).

Statistical analysis was performed using SPSS version 25 (IBM Corp., Armonk, NY, USA). Quantitative data, such as age, are presented in the form of mean and standard deviation. Qualitative data, such as gender, age group, marital status, residence, socioeconomic status, education, occupation, addictions, cancer type, cancer site, cancer grade, clinical stage, and treatment response are presented in the form of frequency and percentages, and the gefitinib and methotrexate groups were compared by applying a chi-square or Fisher’s exact test with a p-value ≤ 0.05 marking significance.

## Results

The mean age of the cancer patients was 49.42 ± 8.11 years in the gefitinib group and 46.67 ± 9.52 years in the methotrexate group. A majority of the patients in the gefitinib group versus the methotrexate group were male (73.0% vs. 82.0%).

In most patients, the type of cancer was new (87.0% vs. 85.0% for the gefitinib group vs. the methotrexate group, respectively; p = 0.684), site of the cancer was the oral cavity (60.0% vs. 64.0%, respectively; p = 0.563), the cancer was grade II (53.0% vs. 57.0%, respectively; p = 0.761), and the clinical stage was IV-B (73.0% vs. 77.0%, respectively; p = 0.514). See Table 1 for a breakdown of these details.

**Table 1:** Cancer characteristics of the study’s head and neck cancer patients

Variables	Gefitinib (%) (n=100)	Methotrexate (%) (n=100)	p-value
Cancer type			
Recurrent	13 (13.0)	15 (15.0)	0.684
New	87 (87.0)	85 (85.0)	
Cancer site			
Oral cavity	60 (60.0)	64 (64.0)	0.563*
Oropharynx	22 (22.0)	26 (26.0)	
Hypopharynx	7 (7.0)	4 (4.0)	
Larynx	5 (5.0)	2 (2.0)	
Nasopharynx	6 (6.0)	4 (4.0)	
Cancer grade			
Grade I	41 (41.0)	36 (36.0)	0.761
Grade II	53 (53.0)	57 (57.0)	
Grade III	6 (6.0)	7 (7.0)	
Clinical stage			
IV-A	27 (27.0)	23 (23.0)	0.514
IV-B	73 (73.0)	77 (77.0)	
*Fisher’s exact test			

In the gefitinib group, 11 patients had partial response (11%), 33 patients had progressive disease (33%), and 52 patients had stable disease (52%). In contrast, the methotrexate group had eight patients with partial response (8%), 40 patients with progressive disease (40%), and 47 patients with stable disease (47%). A total of three patients were lost to follow-up in the gefitinib group, and one patient was lost in the methotrexate group. Only one death was reported in the gefitinib group, and four deaths were reported in the methotrexate group. Statistically, no significant difference was observed for overall responses in the groups (p = 0.368; Table 2).

**Table 2:** Treatment response in study head and neck cancer patients

Outcomes	Treatment		Total, no. & %	p-value
	Gefitinib, no. & %	Methotrexate, no. & %		
Partial response	11	8	19	0.38*
	11.0%	8.0%	9.5%	
Progressive disease	33	40	73	
	33.0%	40.0%	36.5%	
Stable disease	52	47	99	
	52.0%	47.0%	49.5%	
LFU	3	1	4	
	3.0%	1.0%	2.0%	
Death	1	4	5	
	1.0%	4.0%	2.5%	
LFU: Lost to follow-up *Fisher’s exact test				

In recurrent cases, six patients treated with gefitinib showed partial response, whereas no cases of partial response were reported in the methotrexate group (27.3% vs. 0%, respectively). Similarly, the in methotrexate group, there was a significantly higher number of progressive and stable disease reported for the recurrent cases than in the gefitinib group (p = 0.045). In new cases, no significant difference was observed between response and the treatment agents (p = 0.463). Table 3 provides more detail on the response rates.

**Table 3:** Stratification of treatment outcomes in the study’s head and neck cancer patients with respect to type of cancer

		Outcomes					
		Partial response, no. and %	Progressive disease, no. and %	Stable disease, no. and %	LFU, no. and %	Death, no. and %	p-value, no. and %
Recurrent	Gefitinib	6	6	10	–	0	0.045*
		27.3%	27.3%	45.5%	–	0.0%	
	Methotrexate	0	10	11	–	1	
		0.0%	45.5%	50.0%	–	4.5%	
New	Gefitinib	5	27	42	3	1	0.507*
		6.4%	34.6%	53.8%	3.8%	1.3%	
	Methotrexate	8	30	36	1	3	
		10.3%	38.5%	46.2%	1.3%	3.8%	
LFU: Lost to follow-up *Fisher’s exact test							

## Discussion

In the southern regions of Pakistan, head and neck cancers are among the leading cancers and are associated with a high risk of incidence and mortality. The reported incidence of head and neck cancers in Pakistan is 18.74%, and the Global Cancer Observatory of Pakistan reports 10.9% new cases and 11.3% mortality in carcinomas of the lip and oral cavity, 2.5% new cases and 2.1% mortality in carcinoma of the larynx, 1.4% new cases and 0.74% mortality in carcinoma of the hypopharynx, 0.71% new cases and 0.93% mortality in carcinoma of the oropharynx, and 0.43% new cases and 0.42% mortality in carcinoma of the nasopharynx [14,15].

This study compared the efficacy of two commonly used drugs (gefitinib and methotrexate) for the treatment of new or recurrent head and neck cancer. The study hypothesis was that both drugs have equal efficacy in terms of achieving an objective response in new or recurrent head and neck cancers.

The incidence rate of head and neck cancer is alarmingly high in Karachi, where most of this study’s patients lived in urban areas. Some of the more common reasons cited for the higher incidence and mortality rates here are low levels of education, poverty, and addictions, such as common use of smoking, betel nuts, pan, gutka, naswar, alcohol consumption, and so on [16-20]. In this study, commonly reported head and neck carcinomas were in the oral cavity (62.0%), followed by the oropharynx (24.0%), hypopharynx (5.5%), nasopharynx (5.0%), and larynx (3.5%). Similar patterns of head and neck carcinomas were reported in other Pakistani studies, including those from Waqar et al. [20], who reported higher rates of oral cavity carcinoma (58.0%), followed by the hypopharynx (22.1%), nasopharynx (5.0%), oropharynx (5.0%), and larynx (4.0%). Aziz et al. also reported high rates of oral cavity carcinoma (69.0%), followed by larynx (31.0%), and oropharyngeal [21]. It has been observed that the incidence of oral cavity carcinoma is extraordinarily high in Pakistan. The World Health Organization (WHO) Global Cancer Report on Pakistan also notes that oral cavity carcinomas are the most prevalent carcinoma, being the second most common among all types of cancers and first among head and neck cancers [15].

In this study, the overall responses to therapy were compared between gefitinib and methotrexate. In both groups, no patients exhibited a complete response to therapy. In the gefitinib group, there was partial response in 11 (11%) patients, progressive disease in 33 (33%), and stable disease in 52 (52%). In the methotrexate group, there was partial response in eight (8%), progressive disease in 40 (40%), and stable disease in 40 (40%). However, no significant difference was noted in the comparisons of the two groups’ responses to therapy. Similar results have also been reported by other researchers, such as Kushwaha et al. [13], who reported no patients with complete response in the gefitinib and methotrexate groups. In their gefitinib group, the partial response rate was 7.7%, progressive disease was 33.3%, and stable disease was 59.0%, with a control rate of 66.7%. In their methotrexate group, partial response was 5.0%, progressive disease was 42.5%, and stable disease was 52.5%, with a DCR of 57.5%. The rate of survival was 23.1% in their gefitinib group and 10.0% in the methotrexate group [10].

In the current study, six patients treated with gefitinib showed a partial response, but no cases of partial response were reported in the methotrexate group (27.3% vs. 0%, respectively; p = 0.043). Differences in the survival rates seen in Kushwaha et al.’s study [13] as compared to our study are due to the duration of follow-up (27 months in Kushwaha et al.’s study vs. 12 months in our study). Stewart et al. also reported complete response in 1.3% versus 0.7%, partial response in 6.4% versus 3.3%, progressive disease in 31.8% versus 28.3%, and stable disease in 45.2% versus 44.1% for their gefitinib versus methotrexate groups, respectively [22]. All of these studies reported higher rates of response in the gefitinib group than in the methotrexate group, but the difference was statistically not significant.

In the Pakistani community, our analysis reveals that gefitinib has marginally better results than methotrexate in recurrent head and neck cancer. Furthermore, gefitinib has the advantage of being taken orally rather than intravenously, so there is no need for hospitalization or IV cannulation. As a result, in the era of a pandemic, it is more practical to use gefitinib to limit patient exposure.

Literature showed that gefitinib has a different toxicity profile as compared to methotrexate. The most common side effects in gefitinib reported are diarrhea, skin toxicity and oral mucositis. One of the meta-analyses also concluded that for patients with advanced HNSCC, gefitinib cannot prolong the overall survival and progression-free survival or improve overall response rate [23]. In the present research, we only observed diarrhea in 2% of the patients in the methotrexate group. In our setup we have observed good results with gefitinib with minimal side effects that enforced us to design this study. The difference in toxicity profile in our study might be due to genetic makeup or small sample size. Hence, in the Pakistani community, our analysis reveals that gefitinib has marginally better results than methotrexate in recurrent head and neck cancer. Furthermore, gefitinib has the advantage of being taken orally rather than intravenously, so there is no need for hospitalization or IV cannulation. As a result, in the era of a pandemic, it is more practical to use gefitinib to limit patient exposure. The drugs, such as pembrolizumab, nivolumab, and cetuximab, are recommended and are already included in treatment guidelines, but they are extremely expensive and are not provided by the Pakistani government. As a result, drug cost is critical for compliance, particularly in developing countries like Pakistan, where this type of cancer is extremely common. In comparison to these inhibitors, gefitinib is a less expensive option.

Our study has a few limitations, including a short follow-up period, a single-center study, and a small sample size. Future studies should be planned with different tertiary care facilities and inhibitors in mind, and quality of life of patients with advanced or recurrent head and neck cancer should be assessed as well.

## Conclusions

Gefitinib had marginally better results in terms of overall response as compared to methotrexate, specifically in recurrent cases of head and neck cancer. In advanced stage new cases, no significant difference was observed between response of both agents. This benefit for recurrent cases and ease of administration, leading to fewer hospital visits in the COVID-19 era, makes gefitinib superior to methotrexate. The present study had its time constraints, and more in-depth studies should be carried out.
